# Identifying patient profiles to personalize care and improve quality of life in EGPA patients: a national cross-sectional survey

**DOI:** 10.1186/s41687-026-01149-3

**Published:** 2026-07-20

**Authors:** Francesca Romana Torracca, Roberto Padoan, Giacomo Emmi, Lara Bernardi, Maria Rosaria Mollo, Omar Shalby, Fabio Tedone, Davide Cafiero, Eugenia Durante

**Affiliations:** 1 Associazione Pazienti Sindrome di Churg Strauss EGPA, APACS APS ETS, COMO, Italy; 2 Rheumatology Unit, Department of Medicine DIMED, University of Padua, Padova, Italy; 3 Department of Medical, Surgical and Health Sciences, University of Trieste, Trieste, Italy; 4 Centre Hospitalier Universitaire Vaudois, Immunology and Allergy Service, University of Lausanne, Lausanne, Switzerland; 5 Centre for Inflammatory Diseases, Department of Medicine, Monash University, Monash Medical Centre, Melbourne, Australia; 6 GlaxoSmithKline (Italy), Verona, Italy; 7 Helaglobe (Italy), Florence, Italy

**Keywords:** Eosinophilic Granulomatosis with Polyangiitis (EGPA), Quality of life (QoL), Patient-reported outcomes, Disease burden, Patient profiling

## Abstract

**Background:**

Eosinophilic granulomatosis with polyangiitis (EGPA) is a rare, chronic disease that significantly affects quality of life (QoL). Despite improved survival, many patients experience persistent symptoms and complex care needs. Patient-reported data on QoL and perceived care quality remain limited, particularly in Italy. This study aimed to assess health-related QoL and patient-perceived care quality among Italian EGPA patients and to identify distinct patient profiles through cluster analysis to inform personalized, multidisciplinary care strategies.

**Methodology:**

We conducted a cross-sectional, 77-item online survey among adult EGPA patients (self-reported diagnosis) between December 2024 and January 2025. The survey, developed with APACS APS (Associazione Pazienti con Sindrome di Churg-Strauss), was distributed via the SurveyMonkey platform. It included validated instruments: SF-36 for health-related QoL and PACIC (with PACIC-5As) for perceptions of chronic care. Additional demographic, clinical, and disease impact data were collected. Descriptive statistics and group comparisons were performed. SF-36 and PACIC domains were analyzed using principal component analysis (PCA), followed by k-means clustering to identify patient subgroups.

**Results:**

Seventy-two patients completed the survey (mean age 56; 65% female; median disease duration 7 years; 84.7% on biologics; 41.7% on glucocorticoids). SF-36 scores showed moderate QoL impairment (mean PCS 56.3 ± 24.6; MCS 59.3 ± 24.1), with lower QoL among females, divorced/separated individuals, and those with neurological involvement. Paresthesia had the greatest impact, affecting multiple QoL domains and daily functioning (*p* < 0.05). PACIC scores reflected moderate perceptions of care (2.8–3.5), with lowest scores in “Goal Setting” and “Care Coordination.” PACIC-5As scores were low (mean 2.3), indicating suboptimal collaborative care. Cluster analysis identified three profiles: (1) poor QoL with high healthcare engagement (*n* = 12); (2) best QoL and care ratings (*n* = 38); (3) intermediate health status but lowest PACIC scores (*n* = 22), reflecting perceived lack of support. Differences across clusters were statistically significant (*p* < 0.001).

**Conclusions:**

Italian EGPA patients report impaired QoL and only moderate care quality, with notable heterogeneity in experiences. Personalized, multidisciplinary approaches are needed, especially for patients who feel under-supported despite moderate disease activity.

**Supplementary Information:**

The online version contains supplementary material available at https://doi.org/10.1186/s41687-026-01149-3.

## Background

Eosinophilic Granulomatosis with Polyangiitis (EGPA) is a rare systemic necrotizing vasculitis, classified among ANCA-associated vasculitides (AAV), characterized by necrotizing inflammation of small to medium-sized blood vessels, extravascular granulomas, and eosinophilic tissue damage. While any organ can be involved, the lungs, skin, sinuses, cardiovascular system, kidneys, peripheral and central nervous systems, joints, and gastrointestinal tract are most frequently affected [1]. Once associated with high mortality, the disease is now considered chronic and relapsing, owing to advances in immunosuppressive and biologic therapies [2, 3]. However, long-term morbidity remains substantial, as patients often endure persistent symptoms, organ damage, and treatment-related side effects, all of which contribute to significant impairment in health-related quality of life (QoL) [4, 5].

Several studies have demonstrated that patients with AAV experience significantly reduced health-related QoL (HRQoL) compared to the general population, particularly in domains related to physical functioning, fatigue, and emotional well-being [6, 7]. In EGPA the burden of disease is further amplified by frequent involvement of sinonasal tract, lungs, and peripheral nervous system [8]. Symptoms such as chronic rhinosinusitis and peripheral neuropathy may disproportionately impair daily functioning and independence [9, 10].

Although a disease-specific tool such as the AAV-PRO has been developed to more precisely capture HRQoL and disease burden experienced by patients [11–13], generic patient-reported outcome measures (PROMs) such as the Short-Form 36 (SF-36) remain widely employed in this population, offering insights into both physical and mental health dimensions [14].

Beyond health status, assessing the patient’s experience of care has gained growing relevance in chronic, multisystem diseases like EGPA, which require long-term, multidisciplinary management. Tools such as the Patient Assessment of Chronic Illness Care (PACIC) and its PACIC-5As extension evaluate the extent to which care delivery aligns with the Chronic Care Model, including domains such as goal setting, patient activation, and continuity of follow-up [15, 16].

Despite these advances, limited data exist on how EGPA patients perceive both their HRQoL and the quality of chronic care they receive, particularly within specific healthcare systems such as that of Italy. This study aims to address this gap by administering validated PROMs (SF-36 and PACIC) to a cohort of Italian EGPA patients, with the objectives of quantifying disease burden, evaluating care experiences, and identifying distinct patient profiles through cluster analysis. Assessing perceived care alongside disease burden is essential in rare, chronic conditions like EGPA, where patient-centered care and active engagement are key to improving long-term outcomes and quality of life.

## Methods

This was a cross-sectional, web-based survey conducted between December 2024 and January 2025. Adult patients with a self-reported diagnosis of EGPA were recruited through the Italian national patient association APACS (Associazione Pazienti con Sindrome di Churg-Strauss). The survey was developed in collaboration with APACS and disseminated online via the SurveyMonkey platform. All data were collected anonymously via the platform and analyzed in aggregate to ensure participant confidentiality. Only fully completed questionnaires were included in the analysis. No clinical interventions were performed.

The 77-item online questionnaire included three validated patient-reported outcome measures (PROMs) to assess HRQoL and patient experience of care. These included the 36-Item Short Form Health Survey (SF-36), a generic instrument evaluating eight domains of health status (physical functioning, role limitations due to physical health, bodily pain, general health, vitality, social functioning, role-emotional, and mental health), with scores ranging from 0 to 100, where higher values indicate better quality of life. The Patient Assessment of Chronic Illness Care (PACIC), consisting of 20 items across five domains (patient activation, decision support, goal setting, problem solving, and follow-up/coordination), was also administered, with responses scored from 1 to 5 and higher values reflecting a more positive perception of care. Finally, the PACIC-5As, a five-item extension, was used to evaluate the “5As” behavioral counseling framework: Assess, Advise, Agree, Assist, and Arrange.

Additional sections of the survey collected socio-demographic and clinical information, including age, gender, region, education, occupational status, disease duration, manifestations, and treatments. Patients were also asked about the perceived impact of EGPA on daily life, with particular attention to independence, work ability, and social participation.

Descriptive statistics were used to summarize participant characteristics and questionnaire responses. Group comparisons were performed using t-tests or Wilcoxon/Kruskal–Wallis tests, as appropriate, with Bonferroni correction for multiple comparisons. Categorical variables were compared using Chi-squared tests. Spearman’s correlation coefficient was used to assess associations between questionnaire domains.

To identify latent profiles of patients, Principal Component Analysis (PCA) was performed on SF-36 and PACIC domain scores to reduce dimensionality. PCA is a statistical technique used to reduce the complexity of a dataset while preserving as much variance as possible. By transforming original variables (such as individual SF-36 and PACIC scores) into a smaller number of uncorrelated “principal components,” PCA simplifies complex datasets and helps reveal underlying patterns. Components accounting for ≥ 60% of the total variance were retained.

These components were subsequently used as inputs for k-means clustering, an unsupervised machine learning algorithm that partitions patients into distinct, non-overlapping groups (clusters) according to their similarity across the principal component dimensions. The optimal number of clusters was determined to be three, as determined by the elbow method and silhouette analysis, which evaluate both cluster cohesion and separation.

All statistical analyses were performed using Stata 16 (StataCorp, College Station, TX) and Python, with a significance threshold set at *p* < 0.05.

No formal a priori sample size calculation was performed. The project was designed as an exploratory survey in a rare disease population, with recruitment constrained by the number of eligible patients reachable through the national patient association. Although the questionnaire included 77 items, item-level variables were not used as independent predictors in high-dimensional multivariable models. Instead, analyses were based primarily on descriptive statistics, validated questionnaire domain scores, and dimensionality reduction through PCA before clustering.

## Results

Of the 90 survey entries, 72 were fully completed and included in the final analysis, yielding a completion rate of 77%. The main sociodemographic and clinical characteristics of the study population are summarized in Table 1, with additional details provided in Supplementary Table 1. Recipients were predominantly middle-aged (median age 57.5 years, IQR: 49-63.2) and female (65%), with nearly half (48.6%) employed full-time and 65.3% married or cohabiting. A high level of educational attainment was observed, with 41.7% holding a university degree or higher. In a large proportion of cases (61.2%) time since diagnosis exceeded 6 years.

When asked to identify the EGPA manifestations most impactful on daily life, asthma was the most frequently reported (73.6%), followed by nasal polyposis (52.8%) and paresthesias (36.1%). Cardiac involvement was less commonly reported (12.5%). Notably, 33.3% of respondents selected “other” manifestations, which encompassed a broad range of symptoms, including respiratory manifestations (e.g., cough, sputum production, bronchitis, eosinophilic pneumonia), cutaneous (e.g., urticaria, purpura, skin fragility, or ulcers), musculoskeletal (e.g., chronic pain, arthralgia, muscle weakness, osteoporosis), and neurologic manifestations (e.g., peripheral neuropathy, impaired mobility or sensation, tinnitus, hearing loss). Additional reports included sensory disturbances (e.g., anosmia, vasomotor rhinitis), ocular involvement, and generalized fatigue.

Therapeutic regimens at the time of the survey reflected contemporary clinical practice. Most patient (84.7%) reported current or past treatment with biologics, primarily mepolizumab; 41.7% were receiving systemic glucocorticoids, and 29.2% were on conventional immunosuppressants.

The socio-demographic and clinical composition of the clusters was descriptively examined to aid interpretation. Age, education level, occupational status, and time since diagnosis were broadly comparable across clusters. Clusters characterized by poorer QoL showed exploratory descriptive patterns toward a higher proportion of female patients and a greater frequency of paresthesias, relapses, and recent hospitalizations. Given the modest sample size and the small number of patients in some clusters, these findings should be interpreted cautiously and as hypothesis-generating rather than confirmatory.

**Table 1:** Sociodemographic and clinical characteristics of the study population

	*N* = 72
**Age (years)**	58 [49–63]
**Gender**	
Female	47 (65.3)
Male	25 (34.7)
**Years since diagnosis**	
> 10 years	31 (43.1)
6–10 years	13 (18.1)
3–5 years	18 (25.0)
1–2 years	5 (6.9)
< 1 year	5 (6.9)
**Self-reported manifestations**	
Asthma	53 (73.6)
Nasal polyposis	38 (52.8)
Paresthesias	26 (36.1)
Cardiac ments	9 (12.5)
Other	24 (33.3)
**Treatments**	
Biologics	61 (84.7)
Glucocorticoids	30 (41.7)
Immunosuppressive agents	21 (29.2)
Other	13 (18.1)

Data are expressed as absolute numbers (%) and median (IQR)

### Quality of life (SF-36)

**Fig. 1:**
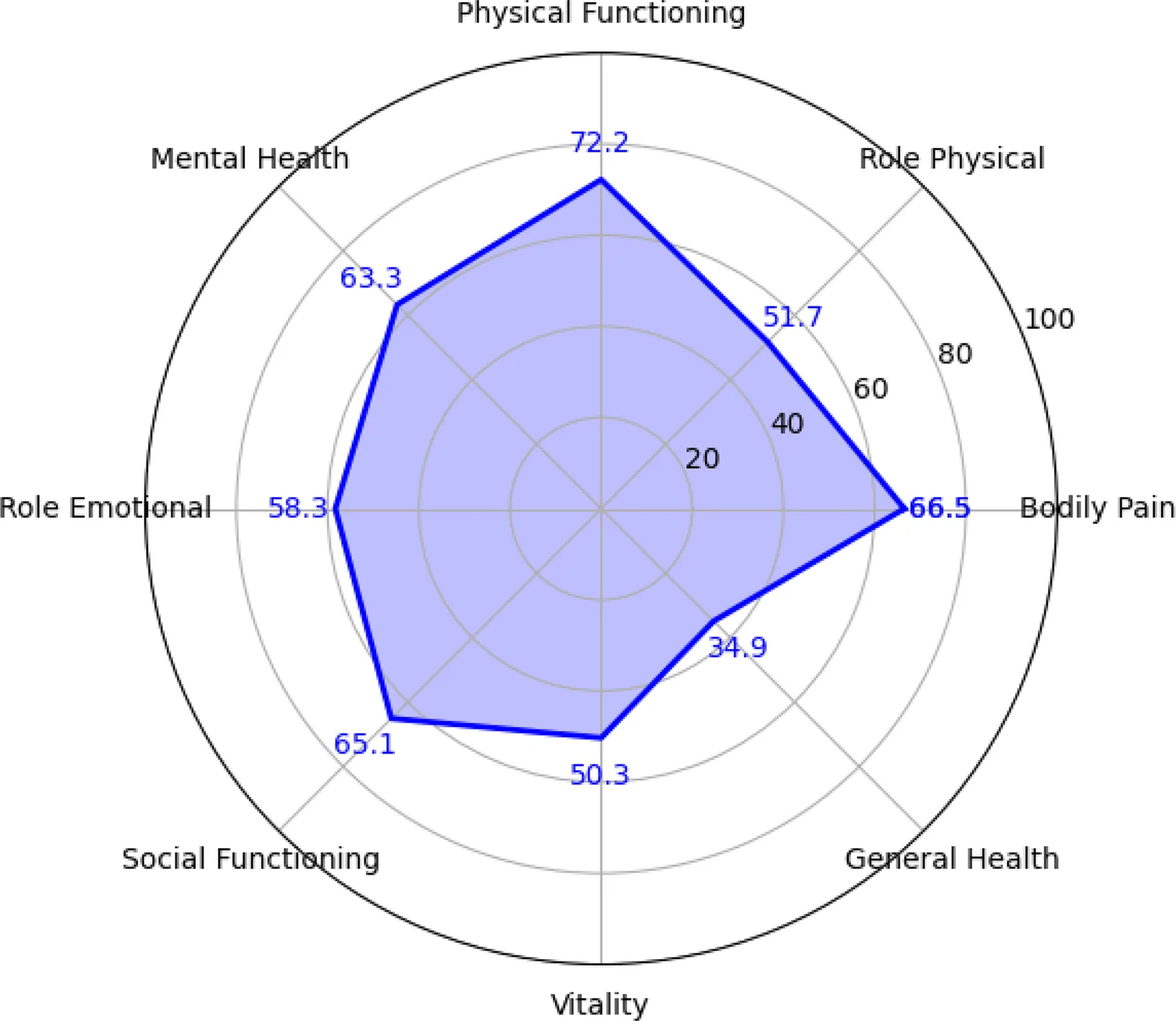
Mean scores for each SF-36 domain among EGPA patients

Overall, patients reported moderate impairment in HRQoL. The mean Physical Component Summary (PCS), which reflects physical functioning, role limitations due to physical health, bodily pain, and general health domains, was 56.3 (SD 24.6). The mean Mental Component Summary (MCS), derived from vitality, social functioning, role emotional, and mental health, was 59.3 (SD 24.1) (Fig. 1).

### Perceived care (PACIC)

**Fig. 2:**
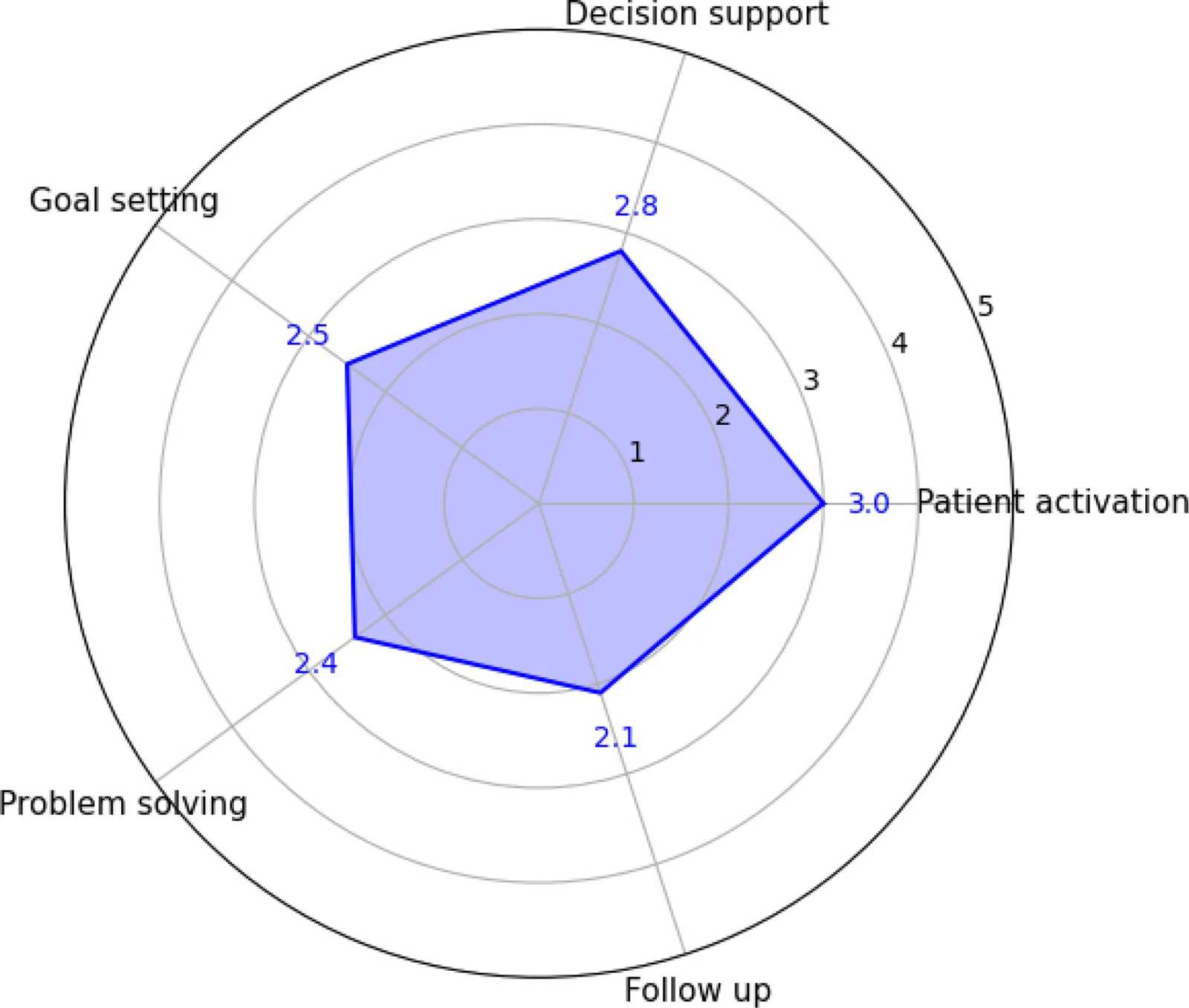
Mean PACIC domain scores in Italian EGPA patients

Patients’ assessments of chronic care quality were moderate. Across the five PACIC domains, mean scores ranged from 2.8 to 3.5 (on a 1–5 scale), with the lowest ratings observed for “Goal Setting” and “Care Coordination” (Fig. 2). Slightly higher values were reported for “Patient Activation” and “Follow-up.” The overall PACIC score in the EGPA cohort was 3.1. Conversely, slightly higher ratings were given for aspects of patient activation and follow-up, but no domain exceeded an average of 3.5, suggesting room for improvement (Fig. 2).

### PACIC-5As

**Fig. 3:**
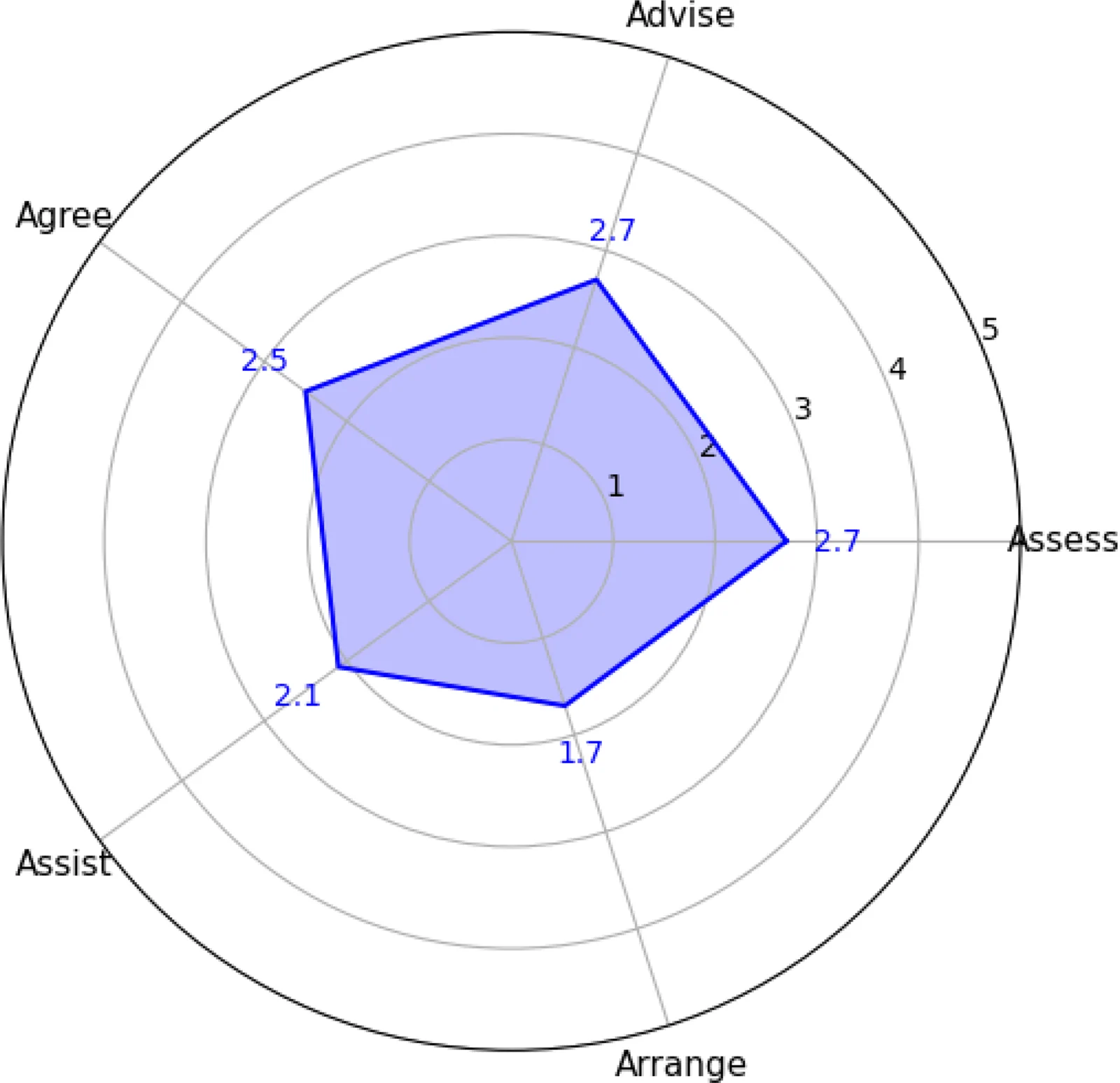
Mean PACIC-5As domain scores in Italian EGPA patients

The PACIC-5As tool yielded a mean overall score of 2.3, reflecting suboptimal integration of collaborative care strategies. As with PACIC, the lowest scores were observed in goal setting and coordination domains (Fig. 3). Better performance was noted in “Assess” and “Assist,” indicating some focus on activation and problem-solving.

### Subgroup analyses

#### Gender

Female patients reported significantly lower SF-36 scores than male patients across multiple domains, including physical functioning, bodily pain, general health, vitality, social functioning, and mental health (all *p* < 0.05; Table 2). No significant differences were observed in the role physical or role emotional domains.

**Table 2:** SF-36 domain scores by gender

	Male (*N* = 25)	Female (*N* = 47)	*p*-value
Physical Functioning	84.8 ± 15.4	65.4 ± 24.3	0.001
Role Physical	60.0 ± 36.1	47.3 ± 45.0	0.235
Bodily Pain	80.0 ± 22.4	59.3 ± 31.2	**0.005**
General Health	42.0 ± 16.1	31.2 ± 17.5	**0.014**
Vitality	64.2 ± 20.2	42.9 ± 20.7	**0.000**
Social Functioning	76.0 ± 18.3	59.3 ± 27.5	**0.009**
Role Emotional	64.0 ± 39.9	55.3 ± 46.3	0.436
Mental Health	70.9 ± 20.2	59.3 ± 18.2	**0.017**

Data are expressed as mean ± SD (standard deviation)

#### Marital status

Quality of life also varied according to marital status (Table 3). Divorced or separated individuals reported significantly lower scores in role physical, bodily pain, and social functioning domains (all *p* < 0.05). Conversely, single or widowed patients showed a non-significant trend toward higher QoL.

**Table 3:** SF-36 domain scores by marital status (median [IQR])

SF-36 domain	Married/Cohabiting (*N* = 47)	Separated/Divorced (*N* = 13)	Widowed (*N* = 3)	Single (*N* = 9)	*p*-value
Physical Functioning	80 [60, 95]	35 [35, 75]	70 [55, 82.5]	85 [80, 90]	0.0931
Role Physical	50 [25, 100]	0 [0, 25]	100 [63, 100]	100 [50, 100]	0.007493
Bodily Pain	75 [50, 100]	38 [25.6, 62.5]	88 [63.9, 97.1]	88 [75, 100]	0.029682
General Health	30 [25.4, 47.5]	25 [15.4, 35]	40 [55.4, 57.5]	40 [35, 50]	0.02568
Vitality	55 [40, 75]	40 [25, 45]	70 [43.7, 70]	50 [50, 70]	0.250834
Social Functioning	63 [63, 81.2]	63 [13.6, 62.5]	100 [69, 100]	63 [63.8, 87.5]	0.036489
Role Emotional	100 [0, 100]	33 [0, 100]	100 [67, 100]	100 [100, 100]	0.456147
Mental Health	68 [52, 84]	56 [44, 64]	64 [52, 76]	52 [44, 72]	0.207912

Data are expressed as median (IQR)

#### Clinical manifestations

**Fig. 4:**
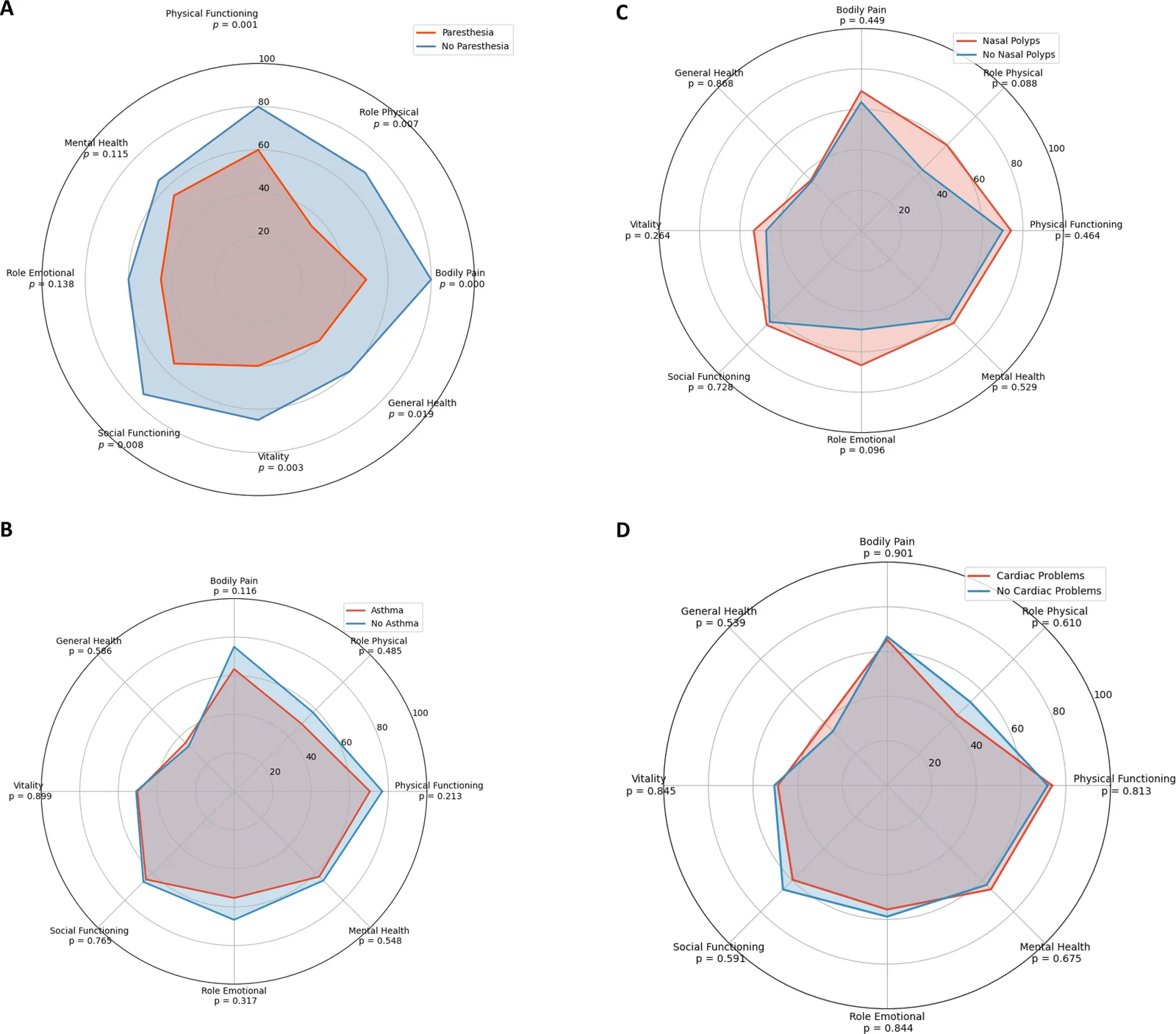
SF-36 Domains: peripheral neurological symptoms (**A**), asthma (**B**), presence of nasal polyposis (**C**) and cardiac involvement (**D**)

Neurological involvement, particularly paresthesias, was associated with the most severe QoL impairment. These patients reported significantly worse scores in physical functioning, role physical, bodily pain, and vitality (all *p* < 0.05; Fig. 4A), as well as greater perceived burden on daily life, including reduced autonomy and work limitations.

Patients reporting asthma (4B) nasal polyposis (4C), cardiac symptoms (4D), did not exhibit significant differences in SF-36 scores. In contrast, those who reported “other” manifestations, such as chronic pain, skin, or ocular issues, had significantly lower physical functioning scores (*p* = 0.017). 

#### Treatment factors

No significant differences in QoL scores were observed according to treatment with glucocorticoids, biologics, or their combination. patients based on glucocorticoids, biologic, or a combination of treatments. The only exception was a lower role-physical score among patients receiving immunosuppressants (*p* = 0.046), which may reflect greater underlying disease severity.

### Perceived disease burden

The perceived disease burden was substantial. Only 36% of men reported no work-related impact, with notably lower rates among women (14.9% reported no impact). Patients with higher educational attainment were more likely to report full independence in daily activities (67.1% vs. 40% in the lower-education group; *p* = 0.023), suggesting a buffering effect of socioeconomic resources.

Neurological involvement was associated with greater burden. Patients with paresthesias were significantly more likely to report loss of autonomy (65.4% vs. 17.4%, *p* < 0.0001), reduced work ability (65.4% vs. 28.3%, *p* = 0.006), and social difficulties (38.5% vs. 15.2%, *p* = 0.017) compared with those without. Similarly, patients reporting “other manifestations” also showed higher rates of work impairment (62.5% vs. 31.3%, *p* = 0.025).

**Fig. 5:**
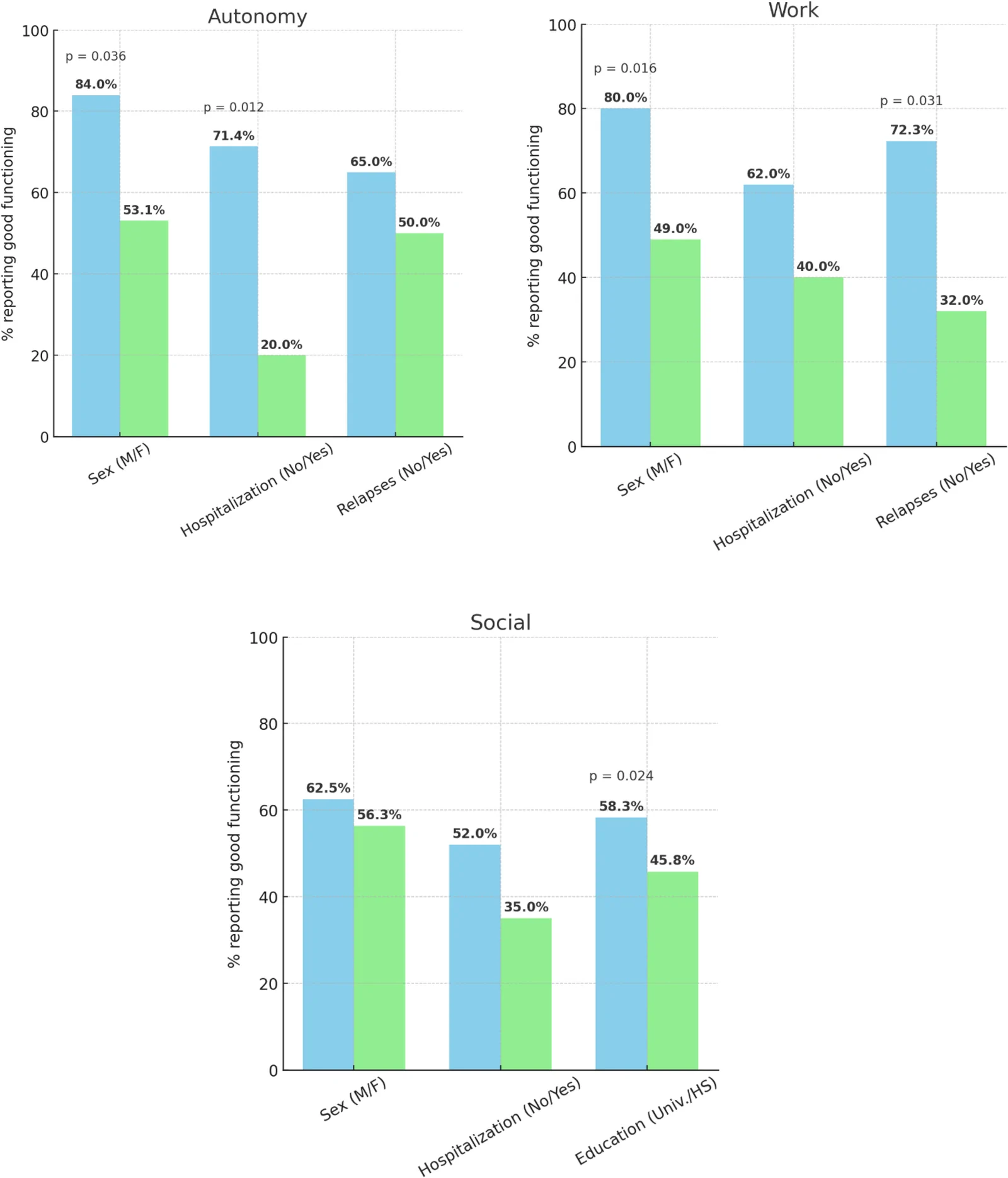
Perceived burden (daily autonomy, work ability, and social impact). Higher values indicate less burden

Perceived burden across the domains of daily autonomy, work ability, and social participation varied significantly between subgroups (Fig. 5). Men reported higher levels of autonomy (84.0% vs. 53.1%, *p* = 0.036) and better work-related functioning (80.0% vs. 49.0%, *p* = 0.016) compared to women. Patients who had not been hospitalized also reported greater autonomy (71.4% vs. 20.0%, *p* = 0.012). Work ability was influenced by disease course, with significantly better functioning among those patients without relapses (72.3% vs. 32.0%, *p* = 0.031). Finally, social functioning was higher among patients with university-level education compared with those with only secondary education (58.3% vs. 45.8%, *p* = 0.024).

### Cluster analysis

Principal component analysis (PCA) identified two primary components explaining 29.7% of the total variance (PC1: 16.4%; PC2: 13.4%).

PC1 reflected overall health status and care quality (SF-36, physical health, bodily pain, vitality, general mental health, and PACIC, advice, goal setting, decision support), while PC2 captured patient activation and engagement (PACIC-5As, agree, assess, patient activation, and problem solving).

**Fig. 6:**
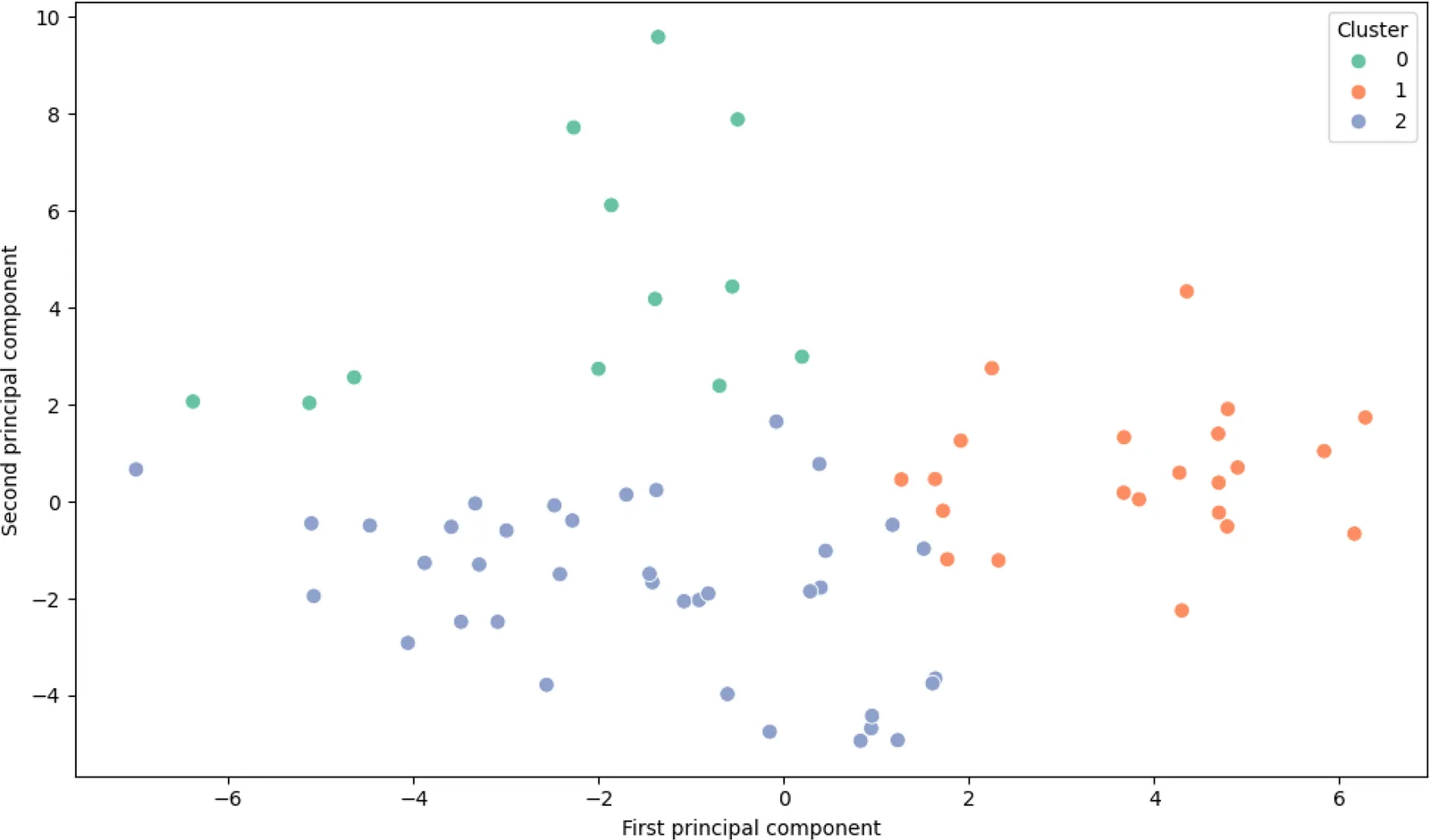
Cluster profiles by summary scores: SF-36 physical/mental and PACIC domains

K-means clustering based on PCA outputs revealed three distinct patient profiles (Fig. 6):

- **Cluster 0 – “High activation**,** poor health” (***n*** = 12 patients)**: Patients reported the lowest physical (mean 24.5 ± 14.0) and mental health (29.5 ± 10.9) scores but paradoxically showed high activation (4.11 ± 0.70) and moderate PACIC scores (3.13 ± 0.78), with intermediate values in the Assist domain (2.55 ± 0.81). This group may represent patients with refractory disease but strong self-management behaviors, possibly facilitated by experience, education, or psychological resilience. Nonetheless, their markedly poor health status underscores the need for intensified clinical support, potentially including rehabilitation and psychological care.
- **Cluster 1 – “Low burden**,** well-supported” (***n*** = 38 patients)**: This cluster showed the best overall health (PCS 77.5 ± 12.3; MCS 77.1 ± 17.1) and the highest PACIC scores (3.29 ± 0.44). Though their Patient activation score (3.38 ± 1.03) was slightly lower than that of Cluster 0, it remained in the high-to-moderate range. These patients likely experience good disease control and coordinated care. This cluster represents resilient, functionally preserved patients who perceive their care as coordinated and effective. They are likely to have a well-controlled disease course, better access to multidisciplinary care, and sufficient relational and informational support. Clinically, this group may benefit from continued standard follow-up with emphasis on maintaining their positive trajectory. Their experience could also inform best practices for patient engagement and care delivery in EGPA.
- **Cluster 2 – “Low support**,** health at risk” (***n*** = 22 patients)**: Despite moderate QoL (Physical Health scores of 54.1 ± 20.5 and Mental Health scores of 58.3 ± 20.9), this group had the lowest PACIC (1.85 ± 0.43) and activation (2.37 ± 0.98) scores, suggesting unmet needs in support and engagement. This profile suggests a potential care gap: despite not being the sickest group clinically, these patients feel unsupported and disempowered. They may be experiencing unmet informational, relational, or emotional needs, which are not adequately addressed by the current healthcare system. If unaddressed, this group risks deterioration in both health outcomes and care engagement. Targeted interventions – such as personalized care plans, structured follow-up, or enhanced patient education – could help bridge the support gap and prevent progression to higher burden.

Significant between-cluster differences were observed across all key variables (all *p* < 0.0001), including physical health, mental health, PACIC overall, patient activation, and the “Assist” subdomain. Figure 6 displays the two-dimensional projection of the sample dataset onto the first and second principal components (PC1 and PC2). Each dot represents an individual respondent. Cluster 0 (green) appears concentrated in the upper-left quadrant, reflecting high PC2 scores (high patient activation, engagement) despite low PC1 (poor QoL), supporting its characterization as “high burden, high engagement”. Cluster 1 (orange) dominates the right side of the plot with high PC1 values (good QoL and positive care perception), aligning with its “resilient and supported” profile. Cluster 2 (blue) spreads across the lower center-left region, characterized by moderate-to-low values on both components, consistent with a “moderate symptoms, low support” phenotype.

Subgroup analyses provide additional context for interpreting these findings, suggesting that HRQoL and care experiences may vary according to selected socio-demographic and clinical characteristics. However, given the modest sample size and the small number of patients in some subgroups, these observations should be interpreted as exploratory and hypothesis-generating. Consistently, clusters characterized by poorer QoL showed descriptive patterns toward a higher proportion of female patients and a greater frequency of paresthesias, relapses, and recent hospitalizations, while age, education, occupational status, and time since diagnosis appeared broadly comparable across clusters. These patterns may help contextualize, but not fully explain, the profiles identified through cluster analysis. Future larger studies should assess whether incorporating subgroup characteristics into cluster-based approaches may support more personalized care strategies. Patients in Cluster 2, in particular, may represent an opportunity for tailored interventions, such as structured follow-up and enhanced collaborative care models.

The clustering underscores heterogeneity in the EGPA patient experience: even among patients with similar clinical statuses, perceptions of health and care can diverge. Table 4 outlines key characteristics of the three clusters, including relative levels of physical/mental health and PACIC scores.

**Table 4:** Summary of key characteristics by cluster

Variable	Cluster 0	Cluster 1	Cluster 2
Sample size (n)	12	38	22
SF-36 Physical Health	24.5 ± 14.0	77.5 ± 12.3	54.1 ± 20.5
SF-36 Mental Health	29.5 ± 10.9	77.1 ± 17.2	58.3 ± 20.9
PACIC Overall Score	3.13 ± 0.78	3.29 ± 0.44	1.85 ± 0.43
Patient Activation	4.11 ± 0.70	3.38 ± 1.03	2.37 ± 0.98
PACIC “Assist” domain	2.55 ± 0.81	2.94 ± 0.70	1.48 ± 0.55

Data are expressed as mean ± SD (standard deviation)

## Discussion

The analysis of this survey provides a comprehensive overview of HRQoL and patient-perceived care among Italian patients living with EGPA, a rare and complex vasculitis. By combining validated PROMs and data-driven cluster analysis, we identified key clinical, psychosocial, and organizational factors shaping patients’ experiences and highlighted persisting gaps in care. While generic instruments such as the SF-36 capture broad health status, disease-specific tools like the AAV-PRO have been developed to better capture vasculitis-related symptoms and could complement generic instruments in future studies [11, 12].

Consistent with previous literature, patients in our cohort reported markedly reduced SF-36 scores across all domains compared to general population norms, confirming the persistent burden of EGPA even in a setting of largely controlled disease [4, 6]. Physical health domains were more impaired than mental health, with fatigue and pain emerging as major limitations. Interestingly, our mean PCS and MCS scores were slightly higher than those reported in earlier AAV studies, which often reported averages in the 40–50 range [7, 8]. This difference may reflect advances in multidisciplinary management and broader access to targeted therapies, particularly biologics, in recent years [7, 17].

Subgroup analyses revealed that female gender and divorced or separated marital status were associated with significantly lower QoL across several domains. This finding is consistent previous studies reporting worse health perceptions among women with AAV [7, 18]. Conversely, single or widowed patients showed a non-significant trend toward higher QoL, suggesting that while the presence of a stable partner may offer protective support, it is not the sole determinant of well-being [19]. These results emphasize the role of psychosocial factors, such as caregiving demands, social roles, and availability of emotional support, on perceived health.

Treatment type did not significantly affect QoL; only patients receiving immunosuppressants had lower role-physical scores, likely reflecting more severe disease. These findings align with those of Cazzador et al. [8] and suggest that pharmacologic treatment alone does not determine QoL, reinforcing the need for a more holistic, patient-centered care approach.

Among clinical manifestations, neurological involvement, especially paresthesias, was strongly associated with impaired quality of life, affecting both physical and psychosocial domains as well as autonomy, work capacity, and social participation. This is consistent with prior evidence that neurologic involvement in AAV disproportionately reduces QoL in AAV [4, 8]. Conversely, sinonasal or cardiac manifestations did not significantly affect SF-36 scores, possibly reflecting the limited sensitivity of generic PROMs in capturing disease-specific symptoms. These results support the integration of symptom-specific instruments in EGPA, such as the AAV-PRO, and when relevant, SNOT-22 for sinonasal symptoms [20] and Asthma Control Test (ACT) for respiratory involvement [21], in both clinical practice and research.

Beyond clinical symptoms, the perceived burden of disease was considerable, particularly in relation to work, independence, and social life. Higher education emerged as a protective factor, associated with greater autonomy, whereas treatment regimen was not systematically linked to perceived burden. These observations echo prior studies reporting high rates of work disability and social limitations in AAV [7], underscoring the importance of care models that extend beyond disease control to include vocational and psychosocial support.

In terms of care quality, PACIC and PACIC-5As scores revealed only moderate alignment with the principles of chronic care, with the lowest ratings observed in the domains of goal setting and care coordination. These findings highlight potential areas for enhancement in structuring collaborative care plans and ensuring continuity across providers. The overall PACIC score (~ 3.1) is comparable to findings in systemic sclerosis (~ 3.0) and type 2 diabetes (~ 2.8–3.2) [22, 23], but substantially lower than in structured telemedicine programs for diabetic foot ulcers (~ 4.4), suggesting that more coordinated and patient-centered approaches could substantially improve care experiences [24].

PACIC-5As scores were particularly low in the “Agree,” “Arrange,” and “Follow-up” domains, though somewhat higher in the “Assess” and “Assist”, suggesting that some elements of activation and problem-solving are present in EGPA care, reflecting partial implementation of patient-centered practices. Results were also in line with PACIC-5As scores reported in obesity (2.29) and osteoarthritis (2.5–2.7), as well as coronary heart disease (2.75) [16, 25, 26]. Collectively, these findings point to a broader need to integrate chronic care principles more fully and consistently into EGPA management.

Cluster analysis further underscored the heterogeneity of patient experiences, revealing three distinct profiles defined by HRQoL and care perceptions. Importantly, these clusters suggest that clinical indicators alone may not predict patient engagement or care quality. Patients in one cluster (Cluster 2) appeared particularly vulnerable to being overlooked in routine follow-up, emphasizing the value of personalized approaches that identify “at risk” subgroups before significant deterioration occurs.

The project has limitations, including the modest sample size, which limits statistical power for subgroup comparisons and precludes definitive inference from high-dimensional item-level analyses. For this reason, the multivariable exploratory analyses were performed on validated domain-level scores and reduced PCA components rather than on all questionnaire items. The clustering results should therefore be interpreted as hypothesis-generating and require validation in larger, independent EGPA cohorts.

Although some descriptive patterns were observed across clusters, particularly regarding sex, paresthesias, relapses, and recent hospitalizations, the sample size limits the ability to draw definitive conclusions about socio-demographic or clinical determinants of cluster membership. The cluster analysis should therefore be interpreted primarily as an exploratory tool to identify patient profiles based on QoL and perceived care experience.

Another limitation is that the “other symptoms” category encompassed a heterogeneous group of conditions. Given that this category was selected by more than 30% of respondents, future research should consider further subcategorization of these symptoms to provide a more nuanced understanding of the EGPA patient experience. However, the study provides one of the most detailed assessment of EGPA patients’ perspectives to date in Italy and offers actionable insights into how care models can evolve to better meet their needs.

### Equity considerations

The findings of this study reveal disparities in health outcomes and access to care among subgroups of EGPA patients, including women, divorced/separated individuals, and those with lower educational attainment. These disparities highlight inequities in disease burden and lived experience that extend beyond clinical manifestations, underscoring the need for explicit equity-focused objectives, in both research and clinical practice to address the unique challenges faced by vulnerable populations.

Future work could aim to:

1. Identify barriers to equitable care: Socioeconomic, geographic, and cultural factors may significantly influence access to care, health literacy, and self-management capacity. For example, patients with higher education tend to report greater autonomy and better health outcomes, highlighting the protective role of socioeconomic resources. This aligns with findings from previous studies that emphasize the impact of socioeconomic status on health disparities in chronic diseases [7, 18, 19].
2. Analyze disparities in health outcomes: Women and divorced/separated individuals in this study reported significantly lower HRQoL across multiple domains. These findings are consistent with previous studies in ANCA-associated vasculitis (AAV) showing worse perceived health and quality of life among women [7, 18]. Psychosocial factors, such as caregiving demands, social isolation, and limited emotional support, are likely contributors to these disparities. Carpenter et al. (2009) also noted that the presence of a supportive partner can positively influence HRQoL, though it is not the sole determinant of well-being [19].
3. Develop targeted interventions: Personalized care strategies should be implemented to address the distinct needs of underserved subgroups. For example, integrating psychosocial support, vocational assistance, and educational resources could improve autonomy, work capacity, and social participation, particularly for patients with lower educational attainment or those reporting high disease burden. Previous studies have highlighted the importance of addressing work disability and social limitations in AAV [7, 8]. Additionally, higher education has been shown to mitigate disease burden by enhancing coping mechanisms and access to resources [7].
4. Integrate equity-focused PRO measures: Generic PROMs like SF-36 and PACIC provide valuable insights but fail to fully capture the specific symptoms contextual social burden associated with EGPA. In particular, these instruments may overlook disease manifestations and life impacts that disproportionately affect certain patient subgroups, such as women or individuals with lower socioeconomic resources. Disease-specific tools, such as AAV-PRO, have been developed to better assess vasculitis-related symptoms [11, 12] offering greater sensitivity to aspects of disease burden that may otherwise remain underrepresented. Furthermore, integrating tools such as SNOT-22 for sinonasal symptoms [20] and Asthma Control Test (ACT) for respiratory involvement [21], alongside measures assessing social participation, occupational functioning, and work disability, could provide a more comprehensive equity-oriented understanding of disparities in patient experiences and outcomes. Such an approach may help identify unmet needs across diverse patient profiles and support the development of more inclusive, patient-centered care strategies.

Embedding equity objectives into future research and care models may contribute to reducing disparities in health outcomes and access to care, ensuring that all EGPA patients, regardless of gender, marital status, or socioeconomic background, receive comprehensive, personalized, and equitable support to improve their quality of life. Addressing these disparities is critical for advancing patient-centered care and optimizing long-term outcomes in EGPA.

## Conclusions

EGPA significantly impairs quality of life and reveals gaps in coordinated patient-centered care. Patients at higher risk, such as those with neurological involvement, women, or limited social support, require particular attention. Identifying and supporting these individuals, even when disease activity appears moderate, is essential.

The cluster analysis was performed to move beyond average QoL and care scores and to identify clinically meaningful patient profiles that may inform future personalized care strategies. The three clusters highlighted different combinations of health status, patient activation, and perceived quality of chronic care, suggesting that EGPA patients may require different types of support despite sharing the same diagnosis. In future studies, these profiles should be prospectively validated in larger and more representative cohorts and tested as a framework for tailoring multidisciplinary interventions. For example, patients in Cluster 2, characterized by moderate health status but low activation and poor perceived care quality, may benefit from structured follow-up, improved care coordination, and patient education. Patients in Cluster 0, characterized by poorer QoL despite higher engagement with care, may require more intensive multidisciplinary assessment and targeted self-management support. Thus, cluster-based approaches may help translate patient-reported outcomes into more actionable care pathways for EGPA.

Personalized, multidisciplinary approaches and systematic use of patient-reported outcomes are key to improving long-term outcomes.

## Supplementary Information

Below is the link to the electronic supplementary material.

Supplementary Material 1

## Data Availability

The data are stored in a database owned by GSK.

## Abbreviations

EGPA: Eosinophilic Granulomatosis with Polyangiitis
QoL: Quality of Life
HRQoL: Health–Related Quality of Life
PROMs: Patient–Reported Outcome Measures
SF–36: Item Short Form Health Survey
PACIC: Patient Assessment of Chronic Illness Care
PACIC–5As: Patient Assessment of Chronic Illness Care–5As Extension
PCA: Principal Component Analysis
ACT: Asthma Control Test
SNOT–22: Sinonasal Outcome Test–22 Items
AAV: ANCA–Associated Vasculitides
AAV–PRO: ANCA–Associated Vasculitis Patient–Reported Outcome
PCS: Physical Component Summary (from SF–36)
MCS: Mental Component Summary (from SF–36)
APACS APS: Associazione Pazienti con Sindrome di Churg–Strauss (Patient Association)
IQR: Interquartile Range
