# Supplementary material for: Identifying patient profiles to personalize care and improve quality of life in EGPA patients: a national cross-sectional survey
Source: J Patient Rep Outcomes. 2026 Jul 20;10:193. doi: 10.1186/s41687-026-01149-3 (PMC13672744; doi:10.1186/s41687-026-01149-3)
Supplement: Supplementary file 1 [file 41687_2026_1149_MOESM1_ESM.docx]

**Supplementary materials**

Socio-demographic and clinical characteristics

| **Variable** | **Frequency** | **Percentage** |
| --- | --- | --- |
| **Education** | | |
| High school diploma | 37 | 51.4% |
| University or postgraduate degree | 30 | 41.7% |
| Middle school | 4 | 5.6% |
| Elementary school | 1 | 1.4% |
| **Employment status** | | |
| Full-time employment | 35 | 48.6% |
| Part-time employment | 9 | 12.5% |
| Retired | 19 | 26.4% |
| Unemployed | 9 | 12.5% |
| **Marital status** | | |
| Married/Cohabitating | 47 | 65.3% |
| Single | 9 | 12.5% |
| Separated/Divorced | 13 | 18.1% |
| Widowed | 3 | 4.2% |
| **Specialist visits frequency/year** | | |
| Less than once | 3 | 4.2% |
| 1–2 times | 30 | 41.7% |
| 3–4 times | 23 | 31.9% |
| More than 4 times | 16 | 22.2% |
| **Hospitalizations (last 12 months)** | | |
| No | 63 | 87.5% |
| Yes | 9 | 12.5% |
| **Relapse frequency** | | |
| None | 46 | 63.9% |
| 1–2 times | 18 | 25.0% |
| 3–4 times | 6 | 8.3% |
| More than 4 times | 2 | 2.8% |

Quality of life and burden of disease

| **Domain** | **Mean** | **SD** |
| --- | --- | --- |
| **SF-36** | | |
| Physical functioning | 72.2 | 23.7 |
| Role physical | 51.7 | 42.9 |
| Bodily pain | 66.5 | 30.3 |
| General health | 34.9 | 17.9 |
| Vitality | 50.3 | 23.1 |
| Social functioning | 65.1 | 26.1 |
| Role emotional | 58.3 | 44.7 |
| Mental health | 63.3 | 19.8 |
| **PACIC 5A** | | |
| Assess | 2.7 | 1.0 |
| Advise | 2.7 | 1.0 |
| Agree | 2.5 | 1.0 |
| Assist | 2.1 | 0.9 |
| Arrange | 1.7 | 0.7 |
| **PACIC** | | |
| Patient activation | 3.0 | 1.2 |
| Decision support | 2.8 | 1.1 |
| Goal setting | 2.5 | 1.0 |
| Problem solving | 2.4 | 1.2 |
| Follow-up | 2.1 | 0.8 |
| Overall PACIC | 2.5 | 0.9 |

Other patient-reported measures

| **Variable** | **Frequency** | **Percentage** |
| --- | --- | --- |
| **Instrumental activity autonomy** | | |
| Completely autonomous | 47 | 65.3% |
| Partially autonomous | 20 | 27.8% |
| Poorly autonomous | 5 | 6.9% |
| **Impact on work ability** | | |
| Yes, significantly | 30 | 41.7% |
| Yes, partially | 26 | 36.1% |
| No | 16 | 22.2% |
| **Changes in social/family habits** | | |
| Yes, partially | 38 | 52.8% |
| Yes, significantly | 17 | 23.6% |
| No | 17 | 23.6% |
